# Role of C_4_ carbon fixation in *Ulva prolifera*, the macroalga responsible for the world’s largest green tides

**DOI:** 10.1038/s42003-020-01225-4

**Published:** 2020-09-07

**Authors:** Dongyan Liu, Qian Ma, Ivan Valiela, Donald M. Anderson, John K. Keesing, Kunshan Gao, Yu Zhen, Xiyan Sun, Yujue Wang

**Affiliations:** 1 State Key Laboratory of Estuarine and Coastal Research, Institute of Eco-Chongming, East China Normal University, Shanghai, 200062 China; 2 The Ecosystems Center, Marine Biological Laboratory, Woods Hole, MA 02543 USA; 3 Woods Hole Oceanographic Institution, Woods Hole, MA 02543-1049 USA; 4 CSIRO Oceans and Atmosphere Research, University of Western Australia Oceans Institute, Indian Ocean Marine Research Centre, Crawley, WA Australia; 5 State Key Laboratory of Marine Environmental Science, Xiamen University, Xiamen, 361005 China; 6 College of Environmental Science and Engineering, Ocean University of China, Qingdao, 266100 China; 7 Muping Coastal Environment Research Station, Yantai Institute of Coastal Zone Research, Chinese Academy of Sciences, Yantai, 264003 China

**Keywords:** Plant ecology, Population dynamics, Light responses, Photosynthesis

## Abstract

Most marine algae preferentially assimilate CO_2_ via the Calvin-Benson Cycle (C_3_) and catalyze HCO_3_^−^ dehydration via carbonic anhydrase (CA) as a CO_2_-compensatory mechanism, but certain species utilize the Hatch-Slack Cycle (C_4_) to enhance photosynthesis. The occurrence and importance of the C_4_ pathway remains uncertain, however. Here, we demonstrate that carbon fixation in *Ulva prolifera*, a species responsible for massive green tides, involves a combination of C_3_ and C_4_ pathways_,_ and a CA-supported HCO_3_^−^ mechanism. Analysis of CA and key C_3_ and C_4_ enzymes, and subsequent analysis of δ^13^C photosynthetic products showed that the species assimilates CO_2_ predominately via the C_3_ pathway, uses HCO_3_^−^ via the CA mechanism at low CO_2_ levels, and takes advantage of high irradiance using the C_4_ pathway. This active and multi-faceted carbon acquisition strategy is advantageous for the formation of massive blooms, as thick floating mats are subject to intense surface irradiance and CO_2_ limitation.

## Abstract (web summary)

Liu et al. present evidence that carbon fixation in *Ulva prolifera* takes place via a combination of C_3_ and C_4_ pathways in combination with the enzyme carbonic anhydrase. The active and multi-faceted carbon acquisition strategy in *U. prolifera* is advantageous for the formation of massive blooms as the thick floating mats are subject to intense surface irradiance and CO_2_ limitation.

## Introduction

The Calvin–Benson cycle (C_3_) is the dominant pathway of carbon fixation in marine algae^1^. Rubisco (ribulose-1, 5-bisphosphate carboxylase), a key C_3_ enzyme responsible for carbon fixation, requires inorganic carbon in the form of CO_2_^2^. However, the demand for CO_2_ in algal photosynthesis is generally higher than CO_2_ concentrations in natural seawaters, e.g., the half-saturation constants for CO_2_ of Rubisco in diatoms are 30–60 μM^3^ but CO_2_ concentrations in natural seawater are only 5–25 μM^4^. Thus, CO_2_ can be an important factor limiting algal proliferation in the ocean.

**Fig. 1:**
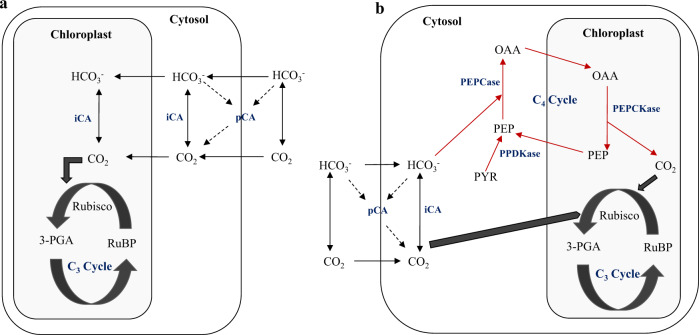
Schematics of two carbon acquisition strategies in marine algae. **a** Transmembrane HCO_3_^−^ is catalyzed by carbonic anhydrase (CA). **b** Transmembrane HCO_3_^−^ is catalyzed by CA and the C_4_ pathway (RuBP 1,2-ribulose diphosphate, 3-PGA 3-phosphoglyceric acid, pCA periplasmic (extra-cellular) CA, iCA intra-cellular CA, PEPCase phosphoenopyruvate carboxylase, PEPCKase phosphoenolpyruvate carboxylase kinase, PPDKase pyruvate orthophosphate dikinase, OAA oxaloacetic acid, PEP phosphoenolpyruvic acid, PYR pyruvic acid).

Since the 1970s there is growing evidence that most species of prokaryotic and eukaryotic algae have developed CO_2_-concentrating mechanisms (CCMs) that enable accumulation of CO_2_ via bicarbonate (HCO_3_^−^) enzymolysis^3,5^. A dominant route in the CCMs is that intra- or extracellular dehydration of HCO_3_^−^ is catalyzed by carbonic anhydrases (CA) to release CO_2_ to increase reactions at the Rubisco site (Fig. 1a). Although traditionally associated with more advanced terrestrial plants, in recent decades a C_4_ or C_4_-like pathway has been discovered in the green alga *Udotea flabellum*^6^ and the marine diatom *Thalassiosira weissflogii*^7,8^, in which transmembrane HCO_3_^−^ is not only catalyzed via CA to generate CO_2_ but also involves C_4_ enzymes—phosphoenolpyruvate carboxylase (PEPCase) and PEPCase kinase (PEPCKase). CO_2_ incorporated in this manner eventually enters the C_3_ cycle to increase reactions at the Rubisco site (Fig. 1b). More recently, a freshwater macrophyte *Ottelia alismoides*, a constitutive C_4_ plant and bicarbonate user, was shown to possess three different CCMs that can operate with the C_4_ pathway in the same tissue, even though Kranz anatomy is absent^9,10^. Theoretically, the joint operation of CCMs and the C_4_ pathway can greatly improve carboxylation efficiency of algal species in the use of HCO_3_^−^, and consequently enhance CO_2_ accumulation at the Rubisco site (Fig. 1b).

*Ulva prolifera*, the main species involved in the massive “green tides” in the Yellow Sea^11^, exhibits remarkable capability for biomass accumulation. *U. prolifera* biomass increases rapidly—more than 60-fold within ~50 days^12,13^. Growth rates in the field were generally higher than 28% per day at temperatures greater than 20 °C^14^, and aerial cover of floating canopies exceed 30,000 km^2^ across the Yellow Sea. Consistent with this, the involvement of the C_4_ pathway in photosynthesis of *U. prolifera* has been suggested as an important mechanism to achieve such rapid biomass accumulation, based on two lines of evidence: (1) gene and enzyme analysis in *U. prolifera* revealed the existence and activity of the C_4_-related enzyme pyruvate orthophosphate dikinase (PPDKase)^15^; and (2) tissue δ^13^C range of *U. prolifera* (−21.9 to −14.9‰) at 16 stations in the Yellow Sea indicated a mix of C_3_ and C_4_ pathways in carbon fixation^16^.

These two lines of evidence, however, are not sufficient to characterize a full C_4_ pathway in *U. prolifera*. That pathway requires the participation of the key C_4_ enzymes, PEPCase and PEPCKase. PEPCase (in the cytoplasm) supports catalysis of carboxylation of phosphoenolpyruvic acid (PEP), which uses HCO_3_^−^ as the inorganic carbon substrate leading to C_4_ compound synthesis. PEPCKase (in the chloroplast) is a decarboxylase, generating CO_2_ and insuring efficient transfer of CO_2_ to Rubisco^17^. PPDKase is responsible for catalyzing the regeneration of PEP in the cytoplasm, but it is not the only way for the C_4_ pathway to acquire PEP^17^. Some researchers even believe that PPDKase might not assist with net CO_2_ fixation but rather has a role in protection against photoinhibition^18^. Therefore, examining the activities of PEPCase and PEPCKase is a necessary step to confirm the existence of C_4_ pathway in *U. prolifera*.

The ^13^C/^12^C distributions in plant tissue records the integrated pattern of photosynthetic carbon acquisition^19,20^; for example, high CO_2_ uptake leads to low δ^13^C values (e.g., <−30‰^21^), whereas high HCO_3_^−^ uptake gives high δ^13^C values (e.g., >−10‰^22^). Therefore, tissue δ^13^C is an important index using to distinguish C_3_ and C_4_ activity. The ^13^C/^12^C values in seaweeds, however, often display a wide range that makes definition of the C_4_ contribution uncertain^20,21^. The variation in the ratios depends on ambient temperature, light, salinity, nutrients and CO_2_ concentrations in seawater^20,22^. Growth and respiration in seaweeds^20,23^ can also lead to discrimination and fractionation of ^13^C/^12^C. As canopies of *U. prolifera* drift in the Yellow Sea, environmental conditions and physiological activity change, forcing variations that may mask clear evidence for C_4_ activity.

To resolve this uncertainty, controlled experiments are needed to examine the correlation between the activities of key enzymes and photosynthetic products to ascertain the activity of C_4_ pathway and/or CA mechanism in *U. prolifera*. Although several environmental factors (e.g., temperature, light, salinity, HCO_3_^−^ and CO_2_ supply, and nutrient availability) may affect the functional expression and activity of CA mechanism and C_4_ pathway, light intensity and *p*CO_2_ are regarded as the most important two under the condition with sufficient nutrient supply^3,5,24^. Massive floating algal mats in nutrient enriched Yellow Sea are not only exposed to strong surface light, but the thick biomass also can reduce the dissolution of CO_2_ from the air and lead to CO_2_ limitation. Consequently, the HCO_3_^−^ systems operated by CA mechanism and/or C_4_ pathway would likely become active.

Here, we describe the results of three outdoor culture experiments designed to examine the daily variations of key C_3_ (Rubisco), CA (extra- and intra-cellular CA) and C_4_-metabolic enzymes (PEPCase, PEPCKase, and PPDKase) in *U. prolifera*. The relationships between the activities of major enzymes and photosynthetic products were analyzed via the corresponding variations in tissue δ^13^C, aiming to identify the participation of C_3_ and C_4_ photosynthetic pathways and CA mechanism in *U. prolifera*. The results exhibited the coexistence of C_3_ and C_4_ pathways and a CA-supported HCO_3_^−^ mechanism in *U. prolifera*. The correlated variations of light intensity and *p*CO_2_ with enzyme activity indicated that the C_4_ pathway was most active under high light but that the CA mechanism became active at low CO_2_ levels. The joint operation of the C_4_ pathway and a CA-supported HCO_3_^−^ mechanism in *U. prolifera* greatly improved efficiency of inorganic carbon fixation and illustrated why massive biomass accumulation can be formed in a short period, when thick floating mats are subject to intense surface irradiance and CO_2_ limitation in the Yellow Sea.

## Results

### The response of key C_3_ and C_4_ enzymes to diurnal sunlight variations

**Fig. 2:**
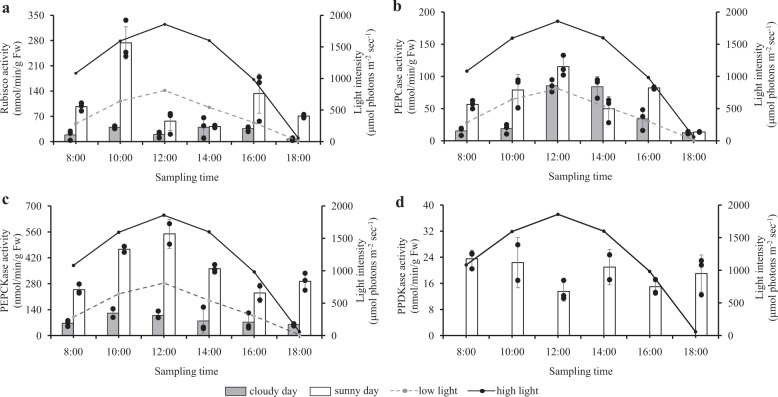
Diurnal patterns of key C3 and C4 enzymes in *U. prolifera*, corresponding to variations in light intensity. **a** The pattern of C_3_ enzyme (Rubisco) on sunny (experiment 1) and cloudy (experiment 2) days. **b**–**d** The pattern of C_4_ enzymes on sunny (experiment 1) and cloudy (experiment 2) days. Each data bar is the mean of three measurements (black dots in each data bar are individual data points from each culture container) and error bars are ±1 standard deviation from the mean. Results of two-way analyses of variance comparing sunny and cloudy days were as follows: Rubisco comparison (all times excluding 18:00), light condition (cloudy/sunny) *F*(1,20) = 33.99, *p* < 0.0001; time *F*(4,20) = 8.43, *p* = 0.0003; light × time interaction *F*(4,20) = 6.25, *p* = 0.002. PEPCase comparison (all times excluding 18:00), light condition *F*(1,20) = 32.74, *p* < 0.0001; time *F*(4,20) = 18.38, *p* < 0.0001; light × time *F*(4,20) = 10.64, *p* < 0.0001. PEPCKase comparison (all times), light condition *F*(1,20) = 152.08, *p* < 0.0001; time *F*(5,20) = 5.56, *p* = 0.002; light × time *F*(5,20) = 2.48, *p* = 0.067. See “Methods” for explanation of comparisons made and any transformations used.

The first two experiments showed that the activity of key C_3_ and C_4_ enzymes were much higher on a sunny day (experiment 1, Fig. 2) than on a cloudy day (experiment 2, Fig. 2). The patterns of C_3_ and C_4_ enzymes differed in response to variations in diurnal sunlight (Fig. 2): mean Rubisco activity was maximal in the morning (10:00 h) but declined significantly from 274 to 57 nmol · min^−1^ · g · fresh weight^−1^ at noon (12:00) under high light intensity (Tukey HSD = 5.327, crit. = 3.541, *p* = 0.001), and stayed low activity between 12:00 and 14:00 h, but increased significantly again from 42 to 143 nmol · min^−1^ · g · fresh weight^−1^ (Tukey HSD = 4.002, crit. = 3.541, *p* = 0.019) between 14:00 and 16:00 under reduced light intensity (Fig. 2a).

**Fig. 3:**
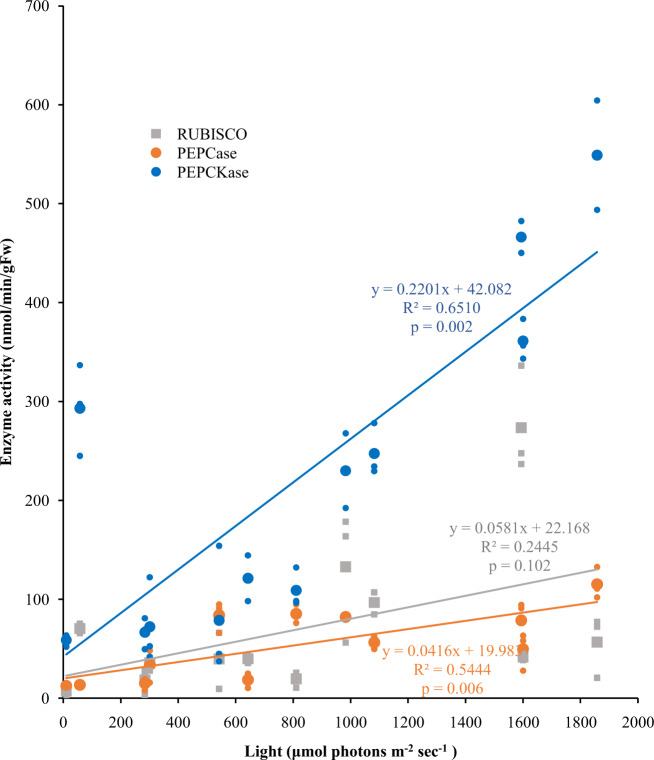
Relationship between irradiance and enzyme activities for Rubisco, PEPCase, and PEPCKase. Means of three measurements (one from each culture container) of enzyme activity for each of 6 time periods in both sunny (experiment 1) and cloudy (experiment 2) days. Equations for regression lines shown are based on the plotted raw data (means: big color dots; individual data points: small color dots). *R*^2^ and *p* values for PEPCKase are for the *χ*^2^ transformed data as reported in the text. The *R*^2^ and *p* value for the raw data were 0.6730 and 0.001, respectively.

In contrast, PEPCase and PEPCKase activities reached maxima at noon, the time of peak irradiation (Fig. 2b, c). PEPCKase activity was significantly higher on the sunny day than on the cloudy day across all time periods from 08:00 to 18:00 (ANOVA, *F*(1,20) = 152.08, *p* < 0.0001, Fig. 2c). PEPCase activity was significantly higher on the sunny day than on the cloudy day at three of the five time periods compared, 08:00, 10:00, and 16:00 (Tukey HSD = 3.634, 5.291, 4.253, respectively, crit. = 3.541, *p* = 0.041, 0.001, and 0.011, respectively, Fig. 2b). PPDKase to catalyze the regeneration of PEP in the C_4_ pathway was only detected on sunny days (Fig. 2d). Regression analysis of enzyme activity against light (Fig. 3) confirm that high activity of C_4_ enzymes (PEPCase [*R*^*2*^ = 0.544, *p* = 0.006] and PEPCKase [*χ*^2^ transformed, *R*^*2*^ = 0.651, *p* = 0.002]) was induced under high irradiance. Rubisco (*R*^2^ = 0.244, *p* = 0.102) did not display a linear relationship with light (Fig. 3). This is consistent with the results of the ANOVA above.

### Relationships between enzyme activities and carbon fixation

**Fig. 4:**
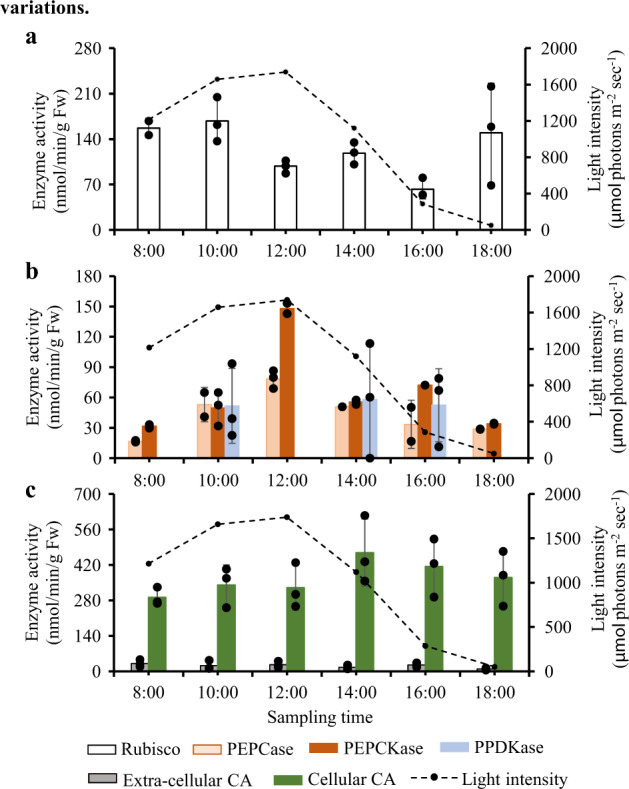
Diurnal patterns of C3 and C4 enzymes and CA in response to sunlight variations. **a** Diurnal pattern of C_3_ enzyme (Rubisco). **b** Diurnal patterns of C_4_ enzyme (PEPCase, PEPCKase, and PPDKase). **c** Diurnal patterns of CA (extracellular and cellular CA). They indicate the activities of C_3_ and C_4_ pathways and CA mechanism, respectively, in response to diurnal sunlight variations. Each data bar is the mean of three measurements (one from each culture container) and error bars are ±1 standard deviation from the mean; black dots in each data bar are individual data points from each culture container.

**Fig. 5:**
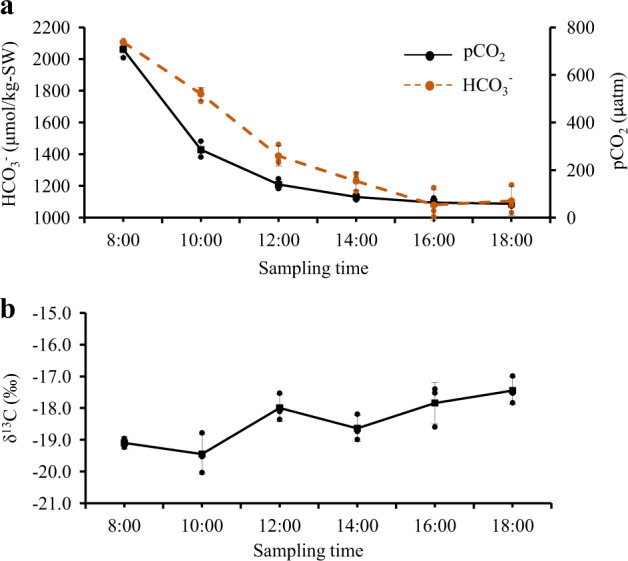
Diurnal variations of *p*CO2 and HCO3^−^ concentrations and tissue δ^13^C. **a** Diurnal variations of *p*CO_2_ and HCO_3_^−^ concentrations in container seawater. **b** Diurnal variations in tissue δ^13^C in *U. prolifera*. Each data point is the mean of three measurements from experiment 3, and the error bars are ±1 standard deviation from the mean (small black and orange dots in each error bar are individual data points from each culture container). Note that the decline in δ^13^C between 08:00 and 10:00 indicates C_3_ dominance, and thereafter, the decrease in δ^13^C is indicative of C_4_ dominance as *p*CO_2_ declines below 200 μatm. Similarly, the decline δ^13^C at 14:00 is associated with an increase in Rubisco (see Fig. 2a).

The third experiment was conducted on a sunny day, aiming to identify the contribution of CO_2_ and HCO_3_^−^ in carbon fixation. The activities of key C_3_ enzyme (Rubisco), C_4_ enzymes (PEPCase, PEPCKase, and PPDKase), and CA enzymes (extra- and intra-cellular CA) and their responses to varying light levels were measured over the course of the day (Fig. 4). Similarly, the variations of *p*CO_2_, HCO_3_^−^, and tissue δ^13^C were also measured (Fig. 5). The intent of this trial was to examine variations in key enzymes that represent the three photosynthetic routes, in relation to variations in photosynthetic products (tissue δ^13^C).

1. Maximal Rubisco activity again occurred in the morning (Fig. 4a), along with rapidly decreased *p*CO_2_ (Fig. 5a), and lowered tissue δ^13^C (Fig. 5b). Photosynthesis during the morning was therefore dominated by a C_3_ pathway supported by sufficient *p*CO_2_ source.
2. PEPCase and PEPCKase activities peaked at noon (Fig. 4b). From 10:00 to 12:00 as irradiance increased, tissue δ^13^C increased 10‰ (Fig. 5b), suggesting a link between C_4_ pathway and carbon fixation using HCO_3_^−^. The activity of PEPCase and PEPCKase and tissue δ^13^C declined markedly at 2 p.m. (Fig. 4b), likely a result of decreased light intensity (Fig. 5b), consistent with the results from experiments 1 and 2, indicating the importance of strong light induction for C_4_ pathway occurrence, rather than low *p*CO_2_.
3. Intra-cellular CA became more active in the afternoon (Fig. 4c) when *p*CO_2_ was below 150 μatm (Fig. 5a). Throughout the experiment, the value of tissue δ^13^C kept increasing after 2 p.m. (Fig. 5b), although the activities of PEPCase and PEPCKase declined markedly (Fig. 4b). Intracellular CA activity was negatively correlated to both *p*CO_2_ (*R*^*2*^ = 0.773, *p* = 0.049) and HCO_3_^−^ concentration (*R*^*2*^ = 0.717, *p* = 0.070), a result that indicated a CA-dominant photosynthetic pathway late in the day when *p*CO_2_ decreased. In contrast, extracellular CA was not active and remained unchanged during the experiment (Fig. 5b), indicating the species mainly used the diffusion of HCO_3_^−^ in cytosol via intracellular CA (Fig. 1b).

These results suggest that *U. prolifera* used a HCO_3_^−^ source via a combination of C_4_ pathways and the CA mechanism in response to changes in irradiation intensity and CO_2_ level.

## Discussion

The results of this study not only demonstrated the coexistence of C_3_ and C_4_ pathways and the CA mechanism in *U. prolifera* but also indicated that the C_4_ pathway catalyzed by PEPCase and PEPCKase were most active in *U. prolifera* photosynthesis under high light. In this regard, the photosynthetic machinery of *U. prolifera* could be similar to that previously described for the green alga *U. flabellum*^6^ and the marine diatom *T. weissflogii*^8^. The CO_2_ delivered to the Rubisco site comes from three sources: the available CO_2_ from seawater, HCO_3_^−^ catalyzed by the CA mechanism, and HCO_3_^−^ catalyzed by PEPCase and PEPCKase via the C_4_ pathway (Fig. 1b). The joint operation of the C_4_ pathway and the CA mechanism greatly improve efficiency of inorganic carbon fixation in *U. prolifera*, and consequently, accelerate the rate of biomass accumulation (Fig. 1b). This multifaceted photosynthetic mechanism has important implications for the ability of this species to grow rapidly when there is high irradiance, and facilitates the formation of the massive floating green tide mats observed in the Yellow Sea^13^.

Studies of the C_4_ pathway in marine algal photosynthesis are very limited, but the results from a few species indicate that the occurrence of a C_4_ pathway and its importance in carbon fixation have distinct species-specific expression. For example, inhibition of PEPCase or PEPCKase could reduce more than 90% of photosynthesis in *T. weissflogii*^8^ and ~50% of photosynthesis in *U. flabellum*^6^. In this study, the range of tissue δ^13^C (−19.1 to −17.4‰) implied an important contribution of HCO_3_^−^ in carbon fixation in *U. prolifera*. We cannot specifically define the contribution of HCO_3_^−^ fixed separately via the CA mechanism versus the C_4_ pathway, but we found that the environmental factors inducing the activity of C_4_ enzymes are different from the CA mechanism. The diurnal modalities of C_4_ enzymes among the three experiments were consistent, although their concentrations were different depending on the ambient environmental conditions at the time of those experiments (Figs. 4 and 5). The repeated modes indicate that the highest PEPCKase activity occurred at maximal light (Figs. 2c and 4b); PEPCKase has a direct correlation with C_4_ acid formation^6^. Previous studies on the diatom *T. pseudonana* also found that light intensity is more important for the C_4_ acid accumulation than low *p*CO_2_ concentrations^25,26^. In this study, tissue δ^13^C showed a marked increase (10‰) at noon, indicating photosynthesis supported by HCO_3_^−^ (Fig. 5) and that the process is operated by C_4_ pathway rather than C_3_ pathway and CA mechanism (Fig. 4). In contrast, HCO_3_^−^ and CO_2_ supply play important roles in modulating activity of C_3_ pathway and CA mechanism^27^. High CO_2_ generally suppresses expression of a high-affinity CCM state^28^, a feature reflected in our result that the C_3_ pathway was active at high *p*CO_2_ concentrations but that the CA mechanism became dominant when *p*CO_2_ was low (Figs. 4 and 5a).

HCO_3_^−^ transport rate and C_4_ activity are energetically costly and require high photon flux densities and sufficient nutrient supply^29,30^ to support synthesis of specific proteins. Excess light energy under strong irradiation however enables ATP to accumulate in the cells and leads to photoinhibition. An active C_4_ pathway at noon indicates that *U. prolifera* has an efficient capability to dissipate excess light energy and ATP in cells. Photosystems I and II (PSI and PSII) are light-harvesting operators, and the energy flux they capture will be quenched and redistributed between PSII/PSI to achieve a balance and avoid irreversible damage to the photosynthetic systems^31^. An induction experiment with *U. prolifera* showed that the electron transport rate (ETR) between PSII/PSI was still high when light intensity reached 800 µmol photons m^−2^ s^−1^ ^32^. Xu and Gao^33^ found that the ETR and net photosynthetic rate of *U. prolifera* remain high and stable even as light intensity increased to 2000 µmol photons m^−2^ s^−1^. These results explain the continuous carbon accumulation of *U. prolifera* exposed to light intensity > 1200 µmol photons m^−2^ s^−1^ for more than 2 h in sunny day experiments, which is about two- or threefold higher than that required to saturate the photosynthetic rate (600 µmol photons m^−2^ s^−1^^33^). PPDKase might play an important role in dissipating excess energy and ATP in cells, since it is responsible for catalyzing the regeneration of PEP in the cytoplasm, where ATP is consumed during PEP formation from pyruvate (Fig. 1b). The basis for this speculation is from the experiments of Haimovich-Dayan et al.^18^ who used RNA-interference to silence the single gene encoding PPDKase in *P. tricornutum* and found that the variations of PPDKase activity hardly affected net CO_2_ fixation but were distinctly involved in dissipating excess energy and ATP in cells. In this study, we only detected PPDKase under sunny conditions, indicating its link with protection against photoinhibition. Based on our study and previous evidence, we propose that, of the three enzymes of C_4_ metabolism, net CO_2_ fixation in *U. prolifera* mainly is determined by PEPCase and PEPCKase, but energy dissipation depends on PPDKase.

Coastal eutrophication and high light supply in the Yellow Sea are important environmental conditions that favor the initiation of C_4_ function of *U. prolifera*. During the summer (June–August) when the *U. prolifera* bloom is forming, dissolved inorganic nitrogen (DIN) concentrations are 10–80 µM near the coast and 1–15 µM offshore^16^, and the average light intensity in surface seawaters is ~1072.2 µmol photons m^−2^ s^−1^ ^34^. In this study, the DIN concentrations in the culture medium were about 30 µM and the daily average light intensity on sunny days in experiments 1 and 3 was 1196 and 1011 µmol photons m^−2^ s^−1^, respectively. These values fit within the range of environmental condition in the Yellow Sea. The *p*CO_2_ supply is limited during the formation of green tide in the Yellow Sea, with a range of 300–450 μatm^35^, and moreover, it can be affected by phytoplankton consumption. Large floating mats formed by *U. prolifera* block air–sea exchange, thus, HCO_3_^−^ uptake and assimilation could become important for rapid biomass accumulation. This was reflected in the tissue δ^13^C (culture samples: −19.5 to −17.4‰; field samples: −21.9 to −14.9‰)^16^.

*Ulva prolifera* assimilates CO_2_ predominately via the C_3_ pathway, takes up HCO_3_^−^ via the CA mechanism at low CO_2_ levels, and takes advantage of high irradiation by deploying the C_4_ pathway. This adaptive and multifaceted carbon acquisition strategy in *U. prolifera* is obviously an advantageous biological approach to support fast biomass accumulation and improve oxygen production in the filaments to sustain respiration for algae while floating in thick mats^36^. The relative contribution of the C_4_ pathway and CA mechanism in utilizing the HCO_3_^−^ system and the environmental thresholds that determine which of these pathways dominate at different stages of bloom development, maturity, and decline need to be further elucidated.

*Ulva prolifera* blooms are initiated from the biomass of thalli dislodged in the intertidal zone where the alga is subject to very high diurnal ranges in temperature and light and can tolerate partial desiccation^37^. Evolutionarily, the expression of C_4_ photosynthesis in *U. prolifera* maybe an adaptive response that enables the species to take advantage of rapidly changing conditions, and which also leads to formation of unusual massive blooms. The ability to harness the benefit of C_4_ photosynthesis might also be a feature of other types of blooming macroalga and warrants investigation.

## Methods

### Culture experiments

Floating specimens of *Ulva prolifera* were collected from the Yellow Sea and, after cleaning epiphytes off using sterilized seawater, the sampled thalli were cultured in a laboratory incubator for a week prior to the outdoor experiments. Fifty grams of fresh thalli were placed into each of three replicate transparent plastic containers (53.5 cm × 39 cm × 32.5 cm), with 40 L of filtered seawater. To maintain ambient seawater temperatures in the containers, they were suspended in an outdoor seawater pond (60 × 100 m) at Muping Coastal Station of Chinese Academy of Sciences, China. Each experiment lasted 1 day from 08:00 to 18:00, without stirring. The first two outdoor culture experiments were carried out in July 2018, including a sunny day culture (experiment 1) and a cloudy day culture (experiment 2). The two experiments were to examine the responses of major enzyme activities of the C_3_ and C_4_ pathways (Rubisco, PEPCase, PEPCKase, and PPDKase) to diurnal variation of light intensity. The details are given below.

Algal sample was collected every 2 h from each of the three culture containers and stored in a liquid nitrogen tank prior to assays of enzyme activities. Light intensity, salinity, and air temperature were monitored during the culture using a light meter (TES-1339R, TES Electrical Electronic Corp., Taiwan), salinometer (S3-Standard kit, Switzerland), and thermometer (PT3003, Anymeter, China), respectively. The initial concentrations of DIN and dissolved inorganic phosphate (DIP) in the culture medium for both the sunny and cloudy day experiments were 34.6 and 0.67 μM, respectively. During experiments 1 and 2, salinity varied from 31.2 to 32.1 and water temperature was not much different on sunny (27.4–32.6 °C) versus cloudy (28.2–31.4 °C) days, but sunlight intensity was much higher on sunny days (57.5–1858 μmol photons m^−2^ s^−1^) than cloudy days (10.2–810 μmol photons m^−2^ s^−1^).

A third outdoor experiment (again with three replicate culture containers as described above) was conducted in August 2019 to define the correlation between major enzyme activities and corresponding photosynthetic products. This experiment was carried out on a sunny day, from 08:00 to 18:00. Samples were collected from each of the three culture containers every 2 h and stored in liquid nitrogen for the assays of enzyme activities and tissue δ^13^C. Meanwhile, the changes of *p*CO_2_ and HCO_3_^−^ were measured in the container seawater, and light, salinity, and air temperature were monitored through the day. The initial concentrations of DIN and DIP in the culture medium were 30.9 and 0.16 μM, respectively. During the third experiment, salinity varied from 33.1 to 33.8, water temperature ranged from 26.2 to 29.6 °C, and sunlight intensity from 50.4 to 1738 μmol photons m^−2^ s^−1^.

### Measurement of the *p*CO_2_ and HCO_3_^−^ concentrations

Twenty-five milliliters of water samples were taken from each of the three culture containers at each 2-h time interval and transferred to a fifty milliliters stoppered polyethylene bottle containing ten milliliters of a hydrochloric acid (HCl) standard solution (0.006 mol L^−1^). After the pH in the water sample was stabilized, HCl standard solution was added into the sample to adjust the pH between 3.40 and 3.90. The added HCl volumes and pH values were recorded and total alkalinity calculated as

$${\mathrm{A}} = \frac{{V_{\mathrm{HCl}} \times c^{\mathrm{HCl}}}}{{V_{\mathrm{W}}}} \times 1000 - \frac{{a_{{\rm{H}}^{+}} \times \left( {V_{\mathrm{W}} + V_{\mathrm{HCl}}} \right)}}{{V_{\mathrm{W}} \times f_{{\rm{H}}^{+}}}}1000,$$

where A represents the alkalinity of the sample (mmol L^−1^); _*c*_^HCl^ represents HCl standard solution (mol L^−1^); *V*_W_ represents water sample volume (mL); *V*_HCl_ represents added HCl volume (mL); $a_{{\rm{H}}^{+}}$ represents the hydrogen ion activity corresponding to the solution pH; and $f_{{\rm{H}}^{+}}$ represents the hydrogen ion activity corresponding to the pH and actual salinity of the solution. All data, including temperature and salinity, were input into CO2SYS^38^, which was used to calculate the *p*CO_2_ and HCO_3_^−^ concentrations.

### Enzyme assays

The frozen algal samples were ground with liquid nitrogen, with ~0.1 g of the ground sample used for enzyme assays. The activities of Rubisco, PEPCase, and PPDKase were assayed using enzyme assay kits provided by Solarbio Life Sciences, China. The activity of PEPCKase was assayed using enzyme assay kits provided by Nanjing Jiancheng Bioengineering Institute, China. One unit of Rubisco, PEPCase, and PEPCKase activity is defined as the amount of enzyme consuming 1 nmol NADH per minute. One unit of PPDKase activity is defined as the amount of enzyme consuming 1 nmol NADPH per minute.

CA activity was assayed according to the method of Wilbur and Anderson^39^. Prior to storage in liquid nitrogen, ~0.1–0.14 g of fresh algal thallus (algal weight) were weighed and then soaked in 2 mL of barbiturate buffer (20 mmol/L, pH = 8.4). Five milliliters of CO_2_-saturated water was injected to initiate the reaction. The reaction mixture was cooled in a 4 °C-water bath. The extracellular CA activity was assayed by measuring the time of catalyzed reaction required for the pH to change from 8.3 to 7.3 (*T*_c_). The same procedure was repeated using 2 mL of barbiturate buffer without algal thallus and the uncatalyzed time required for background pH change from 8.3 to 7.3 was recorded (*T*_0_). The extracellular CA activity was calculated according to the following formula: Enzyme activity (U/g) = ((*T*_0_/*T*_c_ − 1) × 10)/algal weight.

Then, ~0.03–0.04 g of algal thallus was carefully ground in liquid nitrogen and soaked with 2 mL of barbiturate buffer (20 mmol/L, pH = 8.4) for the assay of total CA activity. The total CA activity was measured and calculated using the same method and formula as for extracellular CA activity. The intracellular CA activity was calculated by total CA activity minus extracellular CA activity.

### Tissue δ^13^C measurement

After rinsing with 0.1 M HCl and washing with Milli-Q water, algal samples were freeze-dried and ground. Approximately 0.5–1 mg ground sample was placed into a 4 × 6 mm tin capsule for tissue δ^13^C analysis. The samples were analyzed using an isotope-ratio mass spectrometer (MAT 253, Thermo Scientific, USA) at the Yantai Institute of Coastal Zone Research, Chinese Academy of Sciences. Reference gases were calibrated against International Reference Materials (IAEA-CH6, IAEA-600 and EMA-P1). Results are expressed relative to Vienna PeeDee Belemnite. Replicate measurements of a laboratory standard (acetanilide, Thermo Scientific) analyzed with the samples indicated that analytical errors were <0.1‰ for δ^13^C.

### Statistics and reproducibility

Linear regression analysis was carried out to test the dependence of enzyme activity on irradiance levels using data from both the sunny and cloudy day experiments (experiments 1 and 2). Pearsonʼs *R*^2^ was used to determine the significance of the results. PEPCKase activity was transformed (*χ*^2^) to ensure normality of residuals for the linear regression (Shapiro–Wilk test). Other variables did not require transformation. Two-way analyses of variance (ANOVA, factors = time of day and light condition) was carried out on data from experiments 1 and 2. These analyses compared the level of enzyme activity under sunny and cloudy conditions and between different time periods (three replicates at each time period). Comparisons for PEPCKase were made across all time periods from 08:00 to 18:00, and for Rubisco and PEPCase for time periods from 08:00 to 16:00. Bartletts test was used to check for homogeneity of variances. A square root transformation was required to correct the variance structure for Rubisco and PEPCase. The 18:00 time period was excluded for Rubisco and PEPCase as variance structure could not be corrected if it was included. Tukey’s HSD was used for the pairwise comparisons. Correlation analyses were conducted on parameters measured in the third experiment for the time period from 08:00 to 14:00 (also three replicates) in order to examine the relationships between the activities of key enzymes and photosynthetic products to ascertain the activity of C_4_ and/or CA pathways in *U. prolifera*. All analyses were undertaken using Addinsoft’s statistical software XLSTAT.

### Reporting summary

Further information on research design is available in the Nature Research Reporting Summary linked to this article.

## Supplementary information

Description of Additional Supplementary Files

Supplementary Data 1

Supplementary Data 2

Reporting Summary

## Data Availability

The data supporting the findings of this study and source data of main figures are provided as Supplementary Data.
