# Supplementary material for: Role of C4 carbon fixation in Ulva prolifera, the macroalga responsible for the world’s largest green tides
Source: Commun Biol. 2020 Sep 7;3:494. doi: 10.1038/s42003-020-01225-4 (PMC7477558; doi:10.1038/s42003-020-01225-4)
Supplement: Supplementary file 1 — Description of Additional Supplementary Files [file 42003_2020_1225_MOESM1_ESM.pdf]

Description of Additional Supplementary Files

File Name: **Supplementary Data 1**
Description Data in the main figures

File Name: **Supplementary Data 2**
Description: Raw data in the study
