# Supplementary material for: Role of C4 carbon fixation in Ulva prolifera, the macroalga responsible for the world’s largest green tides
Source: Commun Biol. 2020 Sep 7;3:494. doi: 10.1038/s42003-020-01225-4 (PMC7477558; doi:10.1038/s42003-020-01225-4)
Supplement: Supplementary file 4 — Reporting Summary [file 42003_2020_1225_MOESM4_ESM.pdf]

nature research

Corresponding author(s): Professor Dongyan Liu, East China Normal University, Shanghai, China

Last updated by author(s): Jul 5, 2020

Reporting Summary

Nature Research wishes to improve the reproducibility of the work that we publish. This form provides structure for consistency and transparency in reporting. For further information on Nature Research policies, see Authors & Referees and the Editorial Policy Checklist.

Statistics

For all statistical analyses, confirm that the following items are present in the figure legend, table legend, main text, or Methods section.

n/a	Confirmed	
☐	☒	The exact sample size (n) for each experimental group/condition, given as a discrete number and unit of measurement
☐	☒	A statement on whether measurements were taken from distinct samples or whether the same sample was measured repeatedly
☐	☒	The statistical test(s) used AND whether they are one- or two-sided<br>*Only common tests should be described solely by name; describe more complex techniques in the Methods section.*
☐	☒	A description of all covariates tested
☐	☒	A description of any assumptions or corrections, such as tests of normality and adjustment for multiple comparisons
☐	☒	A full description of the statistical parameters including central tendency (e.g. means) or other basic estimates (e.g. regression coefficient) AND variation (e.g. standard deviation) or associated estimates of uncertainty (e.g. confidence intervals)
☐	☒	For null hypothesis testing, the test statistic (e.g. F, t, r) with confidence intervals, effect sizes, degrees of freedom and P value noted<br>*Give P values as exact values whenever suitable.*
☒	☐	For Bayesian analysis, information on the choice of priors and Markov chain Monte Carlo settings
☒	☐	For hierarchical and complex designs, identification of the appropriate level for tests and full reporting of outcomes
☐	☒	Estimates of effect sizes (e.g. Cohen's d, Pearson's r), indicating how they were calculated

Our web collection on statistics for biologists contains articles on many of the points above.

Software and code

Policy information about availability of computer code

Data collection	no software or code was used in data generation
Data analysis	XLSTAT is a standard statistical software package produced by Addinsoft, a UK company. No unique code was written for this study.

For manuscripts utilizing custom algorithms or software that are central to the research but not yet described in published literature, software must be made available to editors/reviewers. We strongly encourage code deposition in a community repository (e.g. GitHub). See the Nature Research guidelines for submitting code & software for further information.

Data

Policy information about availability of data

All manuscripts must include a data availability statement. This statement should provide the following information, where applicable:

- Accession codes, unique identifiers, or web links for publicly available datasets
- A list of figures that have associated raw data
- A description of any restrictions on data availability

All data analyzed during this study are included in an MS Excel data file as supplementary information. In addition all the replicate data points as well as the means are shown on the graphs that comprise the figures in the paper.

Field-specific reporting

Please select the one below that is the best fit for your research. If you are not sure, read the appropriate sections before making your selection.

☐ Life sciences ☐ Behavioural & social sciences ☒ Ecological, evolutionary & environmental sciences

Ecological, evolutionary & environmental sciences study design

All studies must disclose on these points even when the disclosure is negative.

Study description	The study included three outdoor culture experiments, two were conducted on sunny days and one was on a cloudy day. Each experiment was set up with three replicates and started at 8am and ended at 6pm, to examine diurnal variations of photosynthetic enzymes corresponding to light intensity.<br><br>During the experiments, we sampled every two hours. The treatment factors included biological factors (the C3 and C4 enzymes: Rubisco, PEPCase, PEPCKase, and PPDKase, and carbonic anhydrase), physical factors (air temperature, salinity, light intensity), and chemical factors (pH, pCO2 and HCO3−, tissue 13C).<br><br>The relationships between the activities of enzymes and photosynthetic products were analyzed via the corresponding variations in tissue δ13C, aiming to identify the participation of C3, CA and C4 photosynthetic pathways in Ulva prolifera. The influences of light intensity and pCO2 on the activities of three photosynthetic pathways are discussed to help understanding the function of the three photosynthetic pathways in biomass accumulation of U. prolifera blooms.
Research sample	The target algal species in our study was Ulva prolifera, which is the causative species for the massive floating blooms in the Yellow Sea, China. The species belongs to genus Ulva in Chlorophyta, Ulvophycease and is characterized by hollow tubular filaments with branches, and is widely distributed in the intertidal zone and has a one year life cycle. The species in our experiments was collected from the Qingdao coast, Yellow Sea, China, during the blooming period in July 2018 and August 2019. No manipulations of the algal samples were made except (as stated in the methods) that they were cleaned with sterilized seawater and kept in an incubator for one week prior to the culture experiments.
Sampling strategy	The fresh and healthy thalli of Ulva prolifera were collected from the Qingdao coast, Yellow Sea, China in July 2018 and August 2019. The criteria for healthy thalli is dark green colour and the length of thalli is about 10-15 cm to ensure it is in a good growth status. The sampled thalli were cultured in the incubator for a week prior to the outdoor experiments after cleaning epiphytes off using sterilized seawater, to make sure their physiological condition was stable and similar before each culture experiment.
Data collection	Qian Ma, Yu Zhen and Dongyan Liu were responsible for data collection. During the daily culture experiment, the replicate samples of algal thalli were collected every two hours from culture containers and then stored in a liquid nitrogen tank for assays of enzyme activities. Qian Ma was responsible for enzyme analysis. Light intensity, salinity and air temperature were monitored by Yu Zhen and Dongyan Liu, during the culture using a light meter (TES-1339R, TES Electrical Electronic Corp., Taiwan), salinometer (S3-Standard kit, Switzerland) and thermometer (PT3003, Anymeter, China), respectively. pH, pCO2 and HCO3−were measured and calculated by Yu Zhen. Nutrients were analyzed in Dongyan Liu's laboratory at Eats China Normal Uinversity.
Timing and spatial scale	The timing was dependent on the blooming time of Ulva prolifera in each of the two years. The culture experiments in July 2018: sunny day experiment was conducted on 16th July 2018 and the cloudy day experiment was on 26th July 2018. The culture experiments in August 2019: sunny day on 17 August 2019. All experiments started in the morning (8 am) and ended in the afternoon (6pm). Each experiment had three replicate culture tanks<br><br>Three replicate samples of algal thalli were collected (one sample of multiple thalli from each tank) every two hours and the chemical and physical parameters were measured every two hours to match biological samples.
Data exclusions	No data was excluded from the analyses
Reproducibility	In each experiment, we designed 3 replicates. To make sure the role of light intensity to the C4 pathway of Ulva prolifera, we did sunny day and cloudy day experiments in July 2018 first. After we confirmed the occurrence of the C4 pathway in U. prolifera under maximum light, a sunny day experiment in August 2019 was repeated to acquire the photosynthetic pathways in Ulva prolifera. Three experiments were successful and consistent.
Randomization	There was no randomization process. Each experiment had 3 replicates. The only random component was that the algae sampled form each of the three cultures in each time period was selected at random from the tanks
Blinding	Blinding was not relevant to this study. There was no subjective scoring criteria or measurements used.

Did the study involve field work? ☒ Yes ☐ No

Field work, collection and transport

Field conditions	The only fieldwork involved collecting the algae from the coast for use in the outdoor culture experiments
Location	The Ulva prolifera for the outdoor culture experiments was collected from the Qingdao coastline in the Yellow Sea, China, Latitude 36.10 degrees North, Longitude 120.48 degrees East, in July 2018 and August 2019.
Access and import/export	There was no import or export of samples to or from China where all samples were collected and all experiments were done.

Disturbance | The study removed only floating algae from near the coastline, no other disturbance was made

Reporting for specific materials, systems and methods

We require information from authors about some types of materials, experimental systems and methods used in many studies. Here, indicate whether each material, system or method listed is relevant to your study. If you are not sure if a list item applies to your research, read the appropriate section before selecting a response.

Materials & experimental systems

n/a	Involved in the study
☒	☐ Antibodies
☒	☐ Eukaryotic cell lines
☒	☐ Palaeontology
☒	☐ Animals and other organisms
☒	☐ Human research participants
☒	☐ Clinical data

Methods

n/a	Involved in the study
☒	☐ ChIP-seq
☒	☐ Flow cytometry
☒	☐ MRI-based neuroimaging
